# Subclavian Artery Injury During Lung Tumor Resection

**DOI:** 10.7759/cureus.67846

**Published:** 2024-08-26

**Authors:** Yukio Umeda, Kiyohiko Hagiwara, Shinsuke Matsumoto

**Affiliations:** 1 Cardiovascular and Thoracic Surgery, Gifu Prefectural General Medical Center, Gifu, JPN

**Keywords:** iatrogenic injury, thoracic surgery, lung tumor resection, subclavian artery, vascular injury

## Abstract

An open penetrating injury of the subclavian artery is an extremely rare catastrophic situation in thoracic surgery. We experienced a 57-year-old case of iatrogenic subclavian artery injury during the resection of a lung tumor. The injury occurred during the dissection of the adhesion between the stapling site of the previous bullectomy and the chest wall. Systolic blood pressure dropped below 50 mmHg immediately after the injury. Following primary hemostasis achieved with suture closure, the site of injury was sufficiently exposed and successfully repaired.

## Introduction

Penetrating subclavian artery injuries occur infrequently and constitute less than 2% of all civilian vascular traumas because they are well protected by bony structures at the thoracic outlet [1-3].

There are several cases of subclavian artery injury in the literature, but most of them are blunt injuries associated with fracture of the first rib [4,5] or are iatrogenic non-open penetrating injuries associated with fracture repair [6-8] or catheter insertion [9,10].

On the other hand, open penetrating injuries of the subclavian artery are extremely rare and sometimes lethal. This is because of the difficulty in approach related to its anatomy and the lack of standardized strategies for repair due to its rarity.

We report a case of open penetrating injury of the left subclavian artery during lung tumor resection. In the present case, we performed primary hemostasis with suture closure to control the bleeding, including the retrograde blood flow from the vertebral artery, to avoid ischemia of the basilar artery region. After that, repair of the injured site was carried out following sufficient exposure of both the proximal and distal subclavian arteries. To the best of our knowledge, this is the second successfully treated case of iatrogenic subclavian artery injury encountered in chest surgery reported in the literature.

This successful case provided us with valuable insight into precautions to be taken during primary hemostasis and subsequent repair. We believe that sharing these cases will contribute to the establishment of treatment strategies for these rare injuries.

## Case presentation

**Figure 1:**
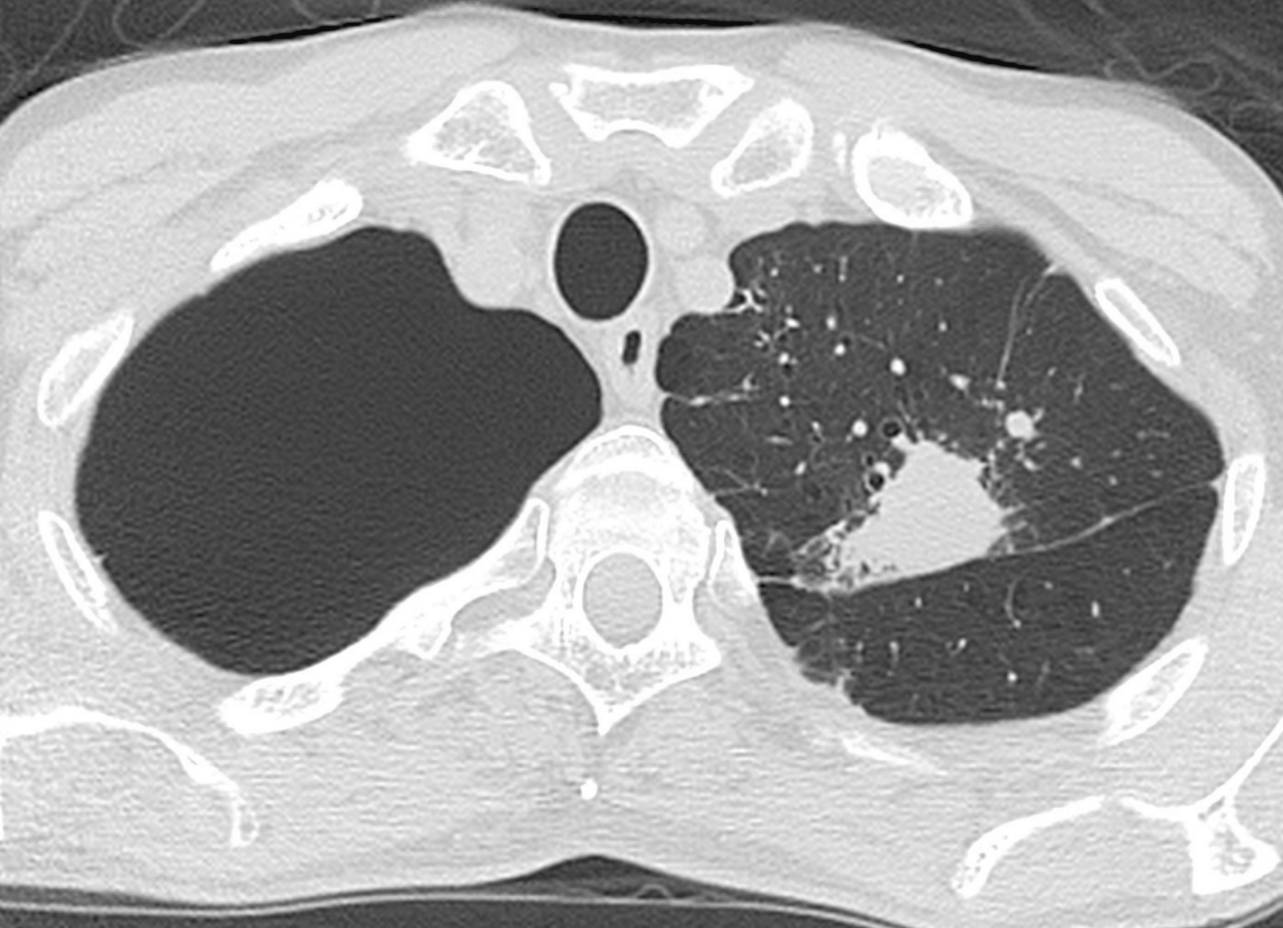
Preoperative CT scan Tumor located in the left upper lobe of the lung.

A 57-year-old man with a history of bullectomy for left pneumothorax was referred to our department for a left lung tumor (lt. S1+2) and planned left upper lobectomy (Figure 1).

A surgical procedure was performed by a certified thoracic surgeon and a certified thoracic and cardiovascular surgeon under epidural and general anesthesia with dual-lumen endotracheal tubes in the right lateral position.

**Figure 2:**
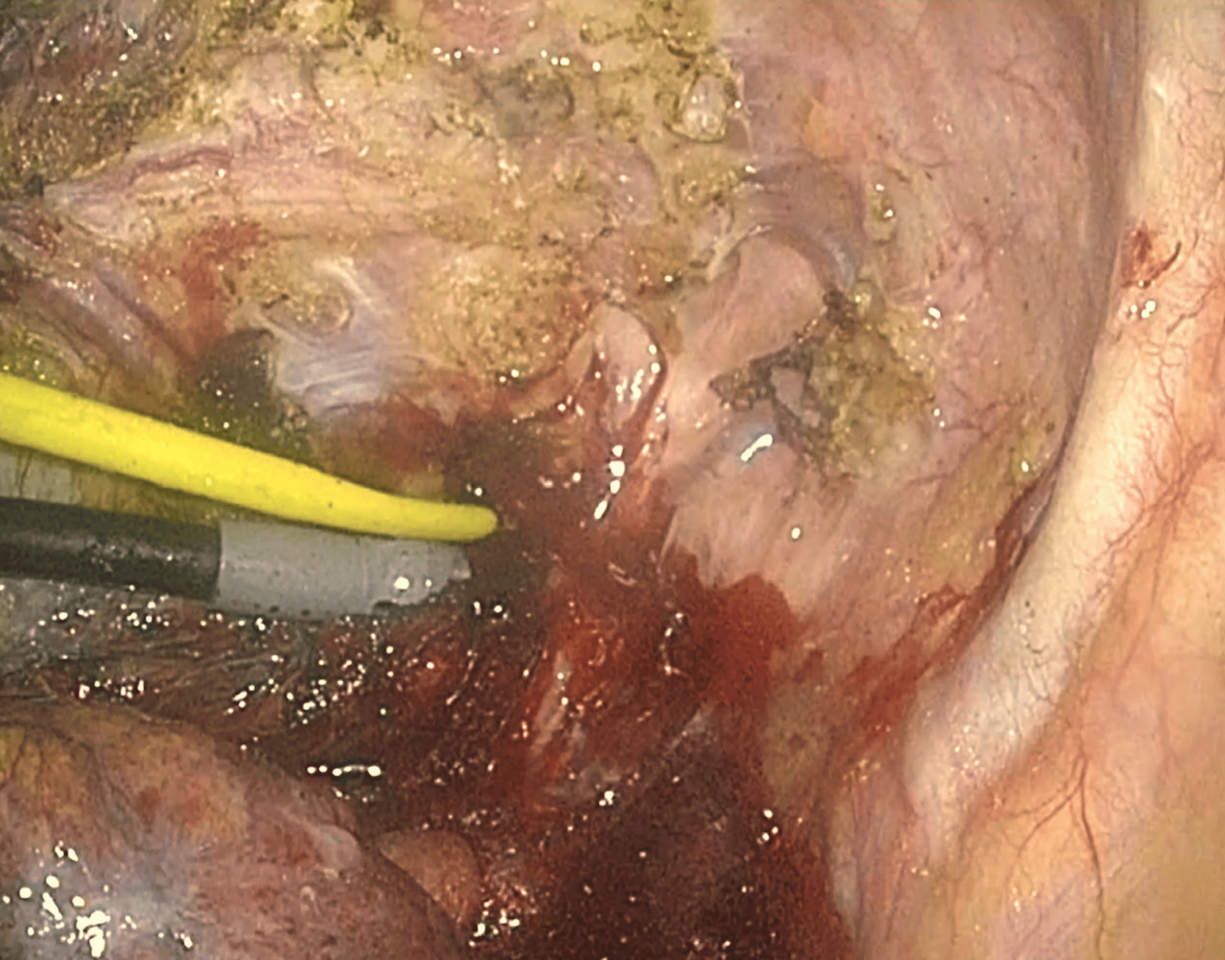
Injured site of the subclavian artery close to the thoracic outlet

Following the observation of the pleural cavity by thoracoscopy inserted through the port placed on the seventh intercostal space (ICS), thoracotomy (8 cm) was made on the fourth ICS. During the dissection of adhesion between the stapling site of the previous bullectomy and the chest wall with electrocautery, massive arterial bleeding was observed (Figure 2, Video 1).

**Video 1:** Injury of the left subclavian artery during dissection of adhesion between the previous stapling site and the chest wall

**Figure 3:**
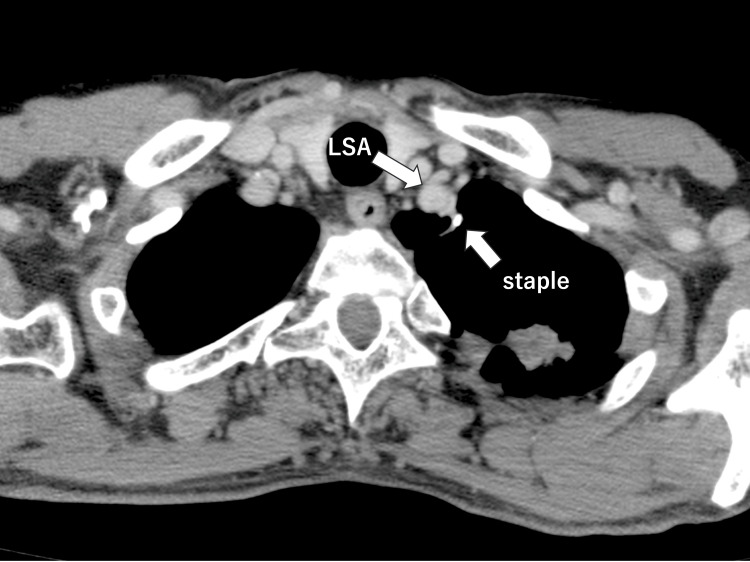
Review of the preoperative CT scan The left subclavian artery (LSA) ran very close to the stapler site of the previous bullectomy.

The injured artery was considered the subclavian artery, and the thoracotomy was extended to 20 cm while controlling the bleeding by compression (Figure 3).

Systolic blood pressure temporarily dropped below 50 mmHg during this period but recovered promptly with adequate compression of the bleeding site after thoracotomy extension and transfusion of fluids and blood products.

The proximal subclavian artery was exposed and clamped by incising the parietal pleura while compressing the bleeding site, but bleeding due to backflow persisted. Because the injured site was close to the thoracic outlet, exposure of the distal subclavian artery was not conceivable under persistent bleeding. Furthermore, the backflow bleeding included a component of retrograde blood flow from the vertebral artery, and there was concern about ischemia of the basilar artery region due to prolonged hemorrhage, so the injured area was suture-closed with 3-0 Prolene (Ethicon Inc., Johnson & Johnson Company, New Brunswick, United States) as primary hemostasis. Following the declamp of the proximal subclavian artery, peripheral arterial pulsation was not as palpable as we considered, so we decided to perform revascularization.

Since complete hemostasis was achieved, the subclavian artery distal to the injured site could be exposed easily and clamped, and the proximal subclavian artery adjacent to the injured area was also dissected and clamped to control backflow from the vertebral artery. After removing the suture used for the primary hemostasis, the injured site was trimmed and repaired with 5-0 Prolene interrupted sutures (Video 2).

**Video 2:** The injured site of the left subclavian artery was sufficiently exposed and repaired following primary hemostasis with suture closure

Then, the left radial artery pulsation could be confirmed. This sequence of procedures for hemostasis and repair was performed without heparinization. A left upper lobectomy was subsequently conducted.

The operative time was 3 hours and 23 minutes, and total blood loss was 1940 ml, requiring transfusion of four units of packed red blood cells and six units of fresh frozen plasma.

The patient was discharged from the hospital on the tenth postoperative day, with no difference in blood pressure in the upper extremities, no neurological abnormality in the left upper extremity, and no significant stenosis of the left subclavian artery confirmed by postoperative enhanced CT scan. A histological examination of the tumor revealed tuberculoma. The patient is doing well 10 years after surgery.

## Discussion

The left subclavian artery originates from the aortic arch behind the left common carotid artery and runs through the superior mediastinal cavity, passing through the posterior to the scalenus anterior muscle and it became the axillary artery at the lateral border of the first rib. While the subclavian artery is a rare site of vascular injury because it runs within the thorax, once injured, its anatomical characteristics make it difficult to achieve prompt proximal and distal control, including the approach. Previous reports have shown that the operative mortality of subclavian artery injury is 5-30% [11-13], especially in the case of open injury, which can quickly develop into hemodynamic disruption if the bleeding is not adequately controlled. In fact, in the present case, systolic blood pressure dropped below 50 mmHg within a minute after injury.

In Japan, the Japan Medical Safety Research Organization has been established to collect, verify, investigate, train, and publish information on medical accidents. The organization is committed to ensuring medical safety and improving the quality of medical care by accumulating and distributing knowledge that is beneficial in developing appropriate preventive strategies for accidents. However, information on individual cases is not disclosed for the protection of personal information [14]. Individual cases of subclavian artery injury during thoracic surgery were sometimes presented at conferences, but a systematical review could not be done. Sharing and refining a sequence of treatment strategies for primary hemostasis and revascularization are essential to salvage patients with intraoperative injuries.

Takase reported a case of subclavian artery injury during a left upper lobectomy at a conference. A cardiovascular surgeon was called to repair the injury, but the cardiovascular surgeon was informed that the site of the injury was unknown upon that call. Then, vascular repair was done under hypothermic extracorporeal circulation. The patient died as a result of prolonged bleeding probably accelerated by hypothermia [15]. As in this report, recognition of the site and mode of injury and sharing of precise information with the surgeon regarding vascular repair are considered crucial. Otherwise, even if extracorporeal circulation with or without hypothermia is not necessary, cardiovascular surgeons who are called to a bailout may use it as a safety strategy beyond what is necessary if the site or mode of injury is unknown.

In the present case, the procedure was performed by a thoracic surgeon and a thoracic and cardiovascular surgeon, and the injured site and the mode of injury were recognized by the surgeon owing the repair, allowing for prompt primary hemostasis and repair.

Primary hemostasis with suture closure allowed for adequate dissection of the subclavian artery both proximal and distal while avoiding impairment of blood flow of the basilar artery. Clamping of the subclavian artery proximal to the vertebral artery bifurcation is not sufficient to control bleeding from the injured site due to certain retrograde blood flow from the vertebral artery. Furthermore, the vertebral artery of the injured side would as a drainage system and might induce ischemia in the basilar artery region, as in subclavian steal syndrome, if this bleeding from the injured site was left untreated. Primary hemostasis with suture closure has been beneficial in eliminating these potential risks.

Endovascular treatment of subclavian artery injuries has been reported to be effective for blunt injuries and non-open penetrating injuries [16-18] but is difficult to consider for open penetrating injuries, where hemodynamics deteriorate rapidly.

Zhu et al. reported the first successfully treated case of iatrogenic subclavian artery injury in the literature [19]. They described a thoracoscopic repair of the subclavian artery injury during thoracoscopic surgery that could not be repaired through the pre-existing port and required a new port.

In fact, the video accompanying their paper showed the difficulty in repairing the injured site at the apex of the thoracic cavity. Inappropriate blind vascular dissection for clamping and deviated technique from basic vascular suturing due to limited needle movement was observed. Their case was fortunate in that blood flow to the upper extremity was maintained, but if blood flow was impaired, further thoracoscopic revascularization would have been impossible to perform.

We considered the extension of the thoracotomy absolutely crucial for primary hemostasis and revascularization and executed it without hesitation.

As for the repair procedure, we intended primary repair because interposition around the thoracic outlet is extremely difficult. In our case, primary hemostasis allowed adequate and safe exposure of the proximal and distal subclavian artery, and proper trimming of the injured site in the bloodless field allowed rapid and reliable primary repair. These procedures were carried out without heparinization, as rapid repair was deemed possible. This was advantageous in reducing the amount of blood loss during the subsequent left upper lobectomy.

Ishibashi reported that a vascular injury requiring a proximal clamp was found in 58 cases (1.5%) among the 3756 cases that underwent thoracic surgery [20]. Also at our institution, vascular injuries requiring proximal clamp were extremely rare, with only three cases (0.2%; present case, axillary artery injury during chest wall tumor resection, and pulmonary artery injury during VATS lobectomy) out of 1412 thoracic surgery cases. Careful preoperative evaluation, consensus on strategies toward injury, and prompt execution at the time of injury are critical. Therefore, once again, we emphasize the importance of global sharing of these extremely rare but potentially catastrophic cases.

## Conclusions

In the present case, hemostasis and revascularization could be promptly and seamlessly carried out for the subclavian artery injury encountered during lung tumor resection because the site and mechanism of injury were shared in real-time with a surgeon owing repair.

Even in cases of massive hemorrhage, we often strive to achieve hemostasis and secure blood flow at the same time. However, performing hemostasis and revascularization in a step-by-step fashion, as in this case, is a safe and reproducible strategy that promises a favorable outcome.
